# Supplementary material for: Light-fuelled freestyle self-oscillators
Source: Nat Commun. 2019 Nov 7;10:5057. doi: 10.1038/s41467-019-13077-6 (PMC6838320; doi:10.1038/s41467-019-13077-6)
Supplement: Supplementary file 1 — Supplementary Information [file 41467_2019_13077_MOESM1_ESM.pdf]

Supplementary Information for

Light driven freestyle self-oscillators

Zeng et al.

Supplementary Figures.

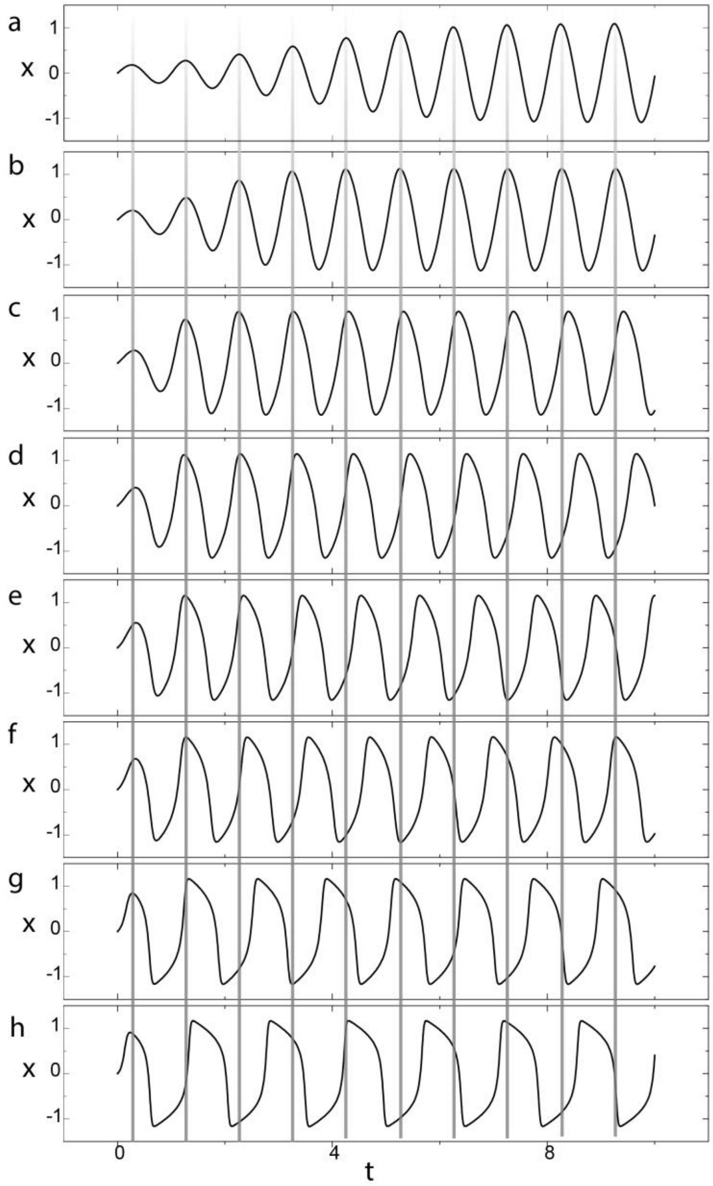

Supplementary Figure 1 | Self-oscillating evolution upon increasing delay. Numerical solutions to the equation, $\ddot{x} - (\sigma\omega_o^2 - \varsigma - \eta x^2)\dot{x} + \omega_o^2 x = 0$, where set $\varsigma = 0.1, \omega_0 = 2\pi$, for initial conditions: $\dot{x}(0) = 1, x(0) = 0$. These show the sequence of elongated oscillating periodicity and reshaping wave front when the delay σ increases as, **(a)** $\sigma\omega_o^2 = 1, \eta = 3$, **(b)** $\sigma\omega_o^2 = 2, \eta = 6$, **(c)** $\sigma\omega_o^2 = 4, \eta = 12$, **(d)** $\sigma\omega_o^2 = 6, \eta = 18$, **(e)** $\sigma\omega_o^2 = 8, \eta = 24$, **(f)** $\sigma\omega_o^2 = 10, \eta = 30$, **(g)** $\sigma\omega_o^2 = 15, \eta = 45$, **(h)** $\sigma\omega_o^2 = 20, \eta = 60$. The numerical calculations are performed with Matlab R2013a.

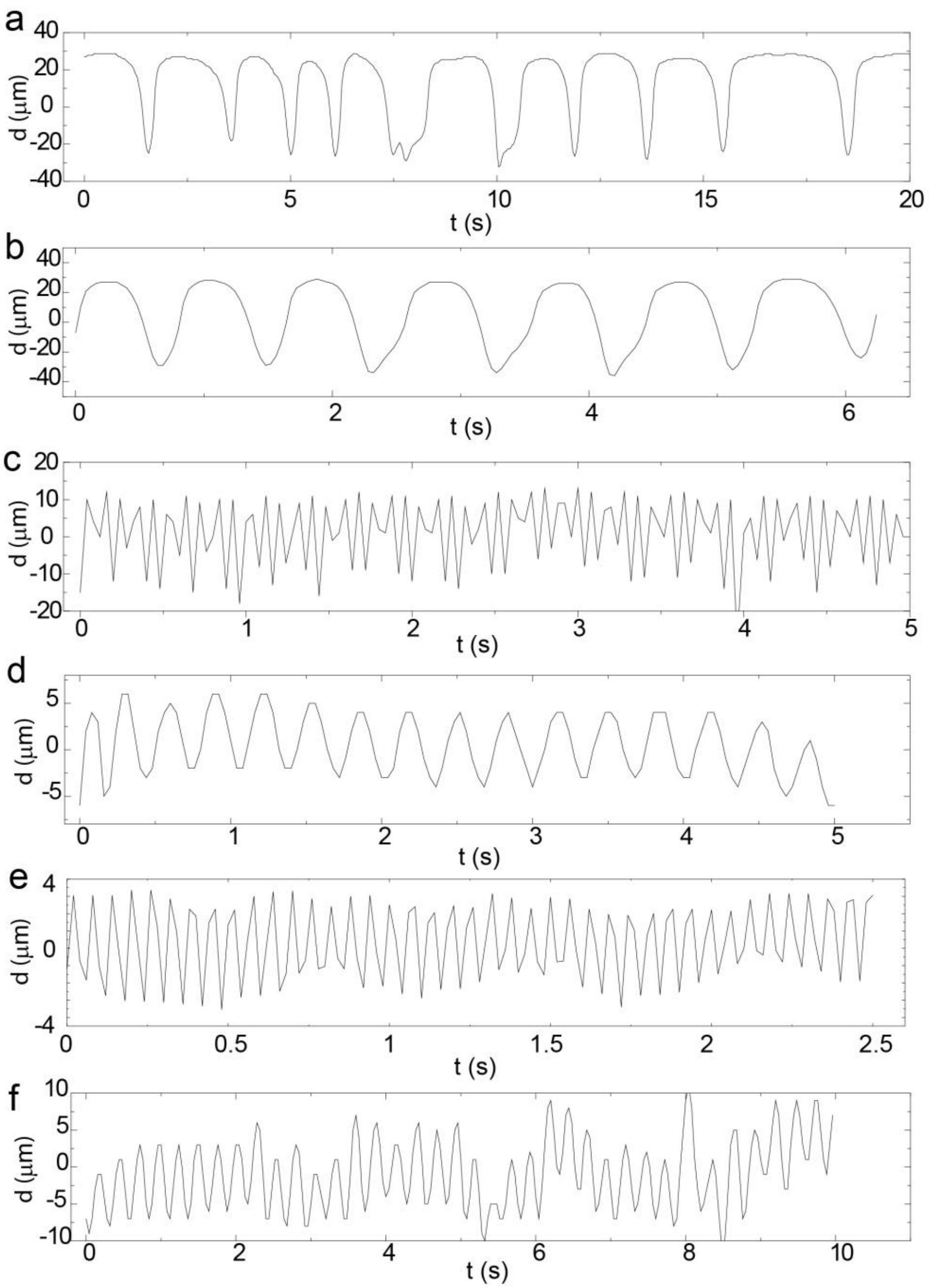

Supplementary Figure 2 | Observation of contracting-expanding mode self-oscillation. (a-f) A set of oscillation observed in an LCN cantilever with contracting-expanding mode. The fibre-like LCN is about 100 μm in diameter, excited upon a focused 488 nm laser beam (100 mW, focused by 10× objective, NA: 0.25).

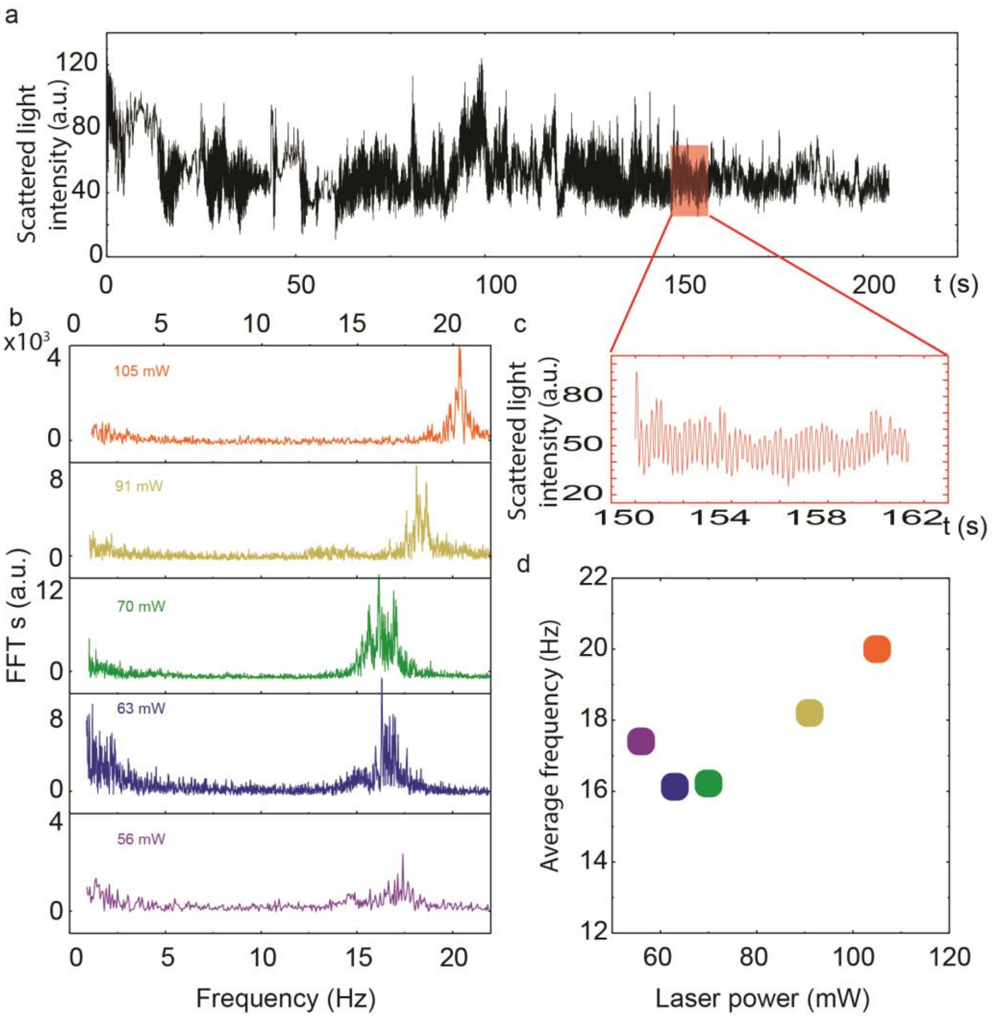

Supplementary Figure 3 | Frequency analysis of contraction-expansion oscillation mode. (a) A typical self-oscillation event recorded by measuring the scattered light intensity from the tip of the LCN during the oscillation. Laser power: 125 mW. A zoom-in image is given in **(c)**. **(b)** Fourier transform at the frequency domain for self-oscillations upon identical excitation beam position but varying the laser power. **(d)** Evolution of self-oscillation frequency along with increase of laser power. The fiber-like LCN is about 100 μm in diameter, and excited with a 488 nm laser beam focused by 10× objective (NA: 0.25).

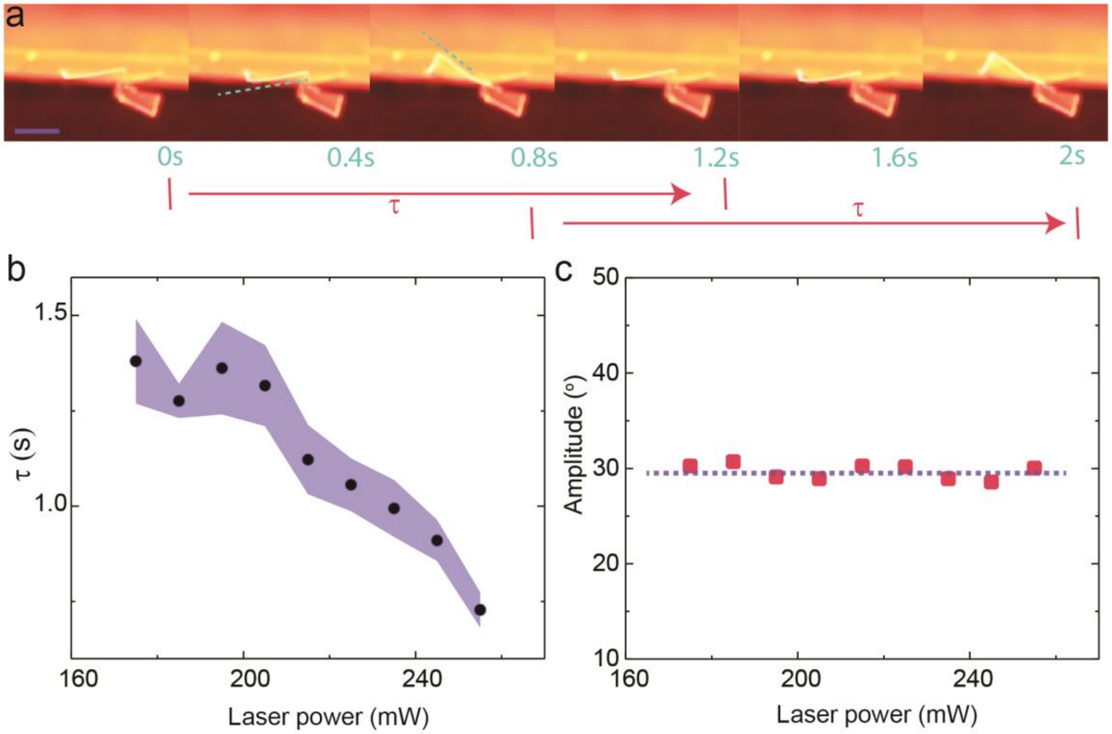

Supplementary Figure 4 | Frequency analysis on an LCN cantilever with twisting mode. (a) A series of photographs showing cyclic twisting oscillation, with arrows indicating the periodicity τ of each oscillating cycle. **(b)** Change of oscillating periodicity upon irradiating with different laser power. Error bars indicate standard deviation of $n > 10$ oscillation periods. **(c)** The oscillation amplitude remains constant with increasing laser power. The size of the LCN is of $3\times4.5\times0.05mm^3$, and it is excited with a non-focused 488 nm laser beam.

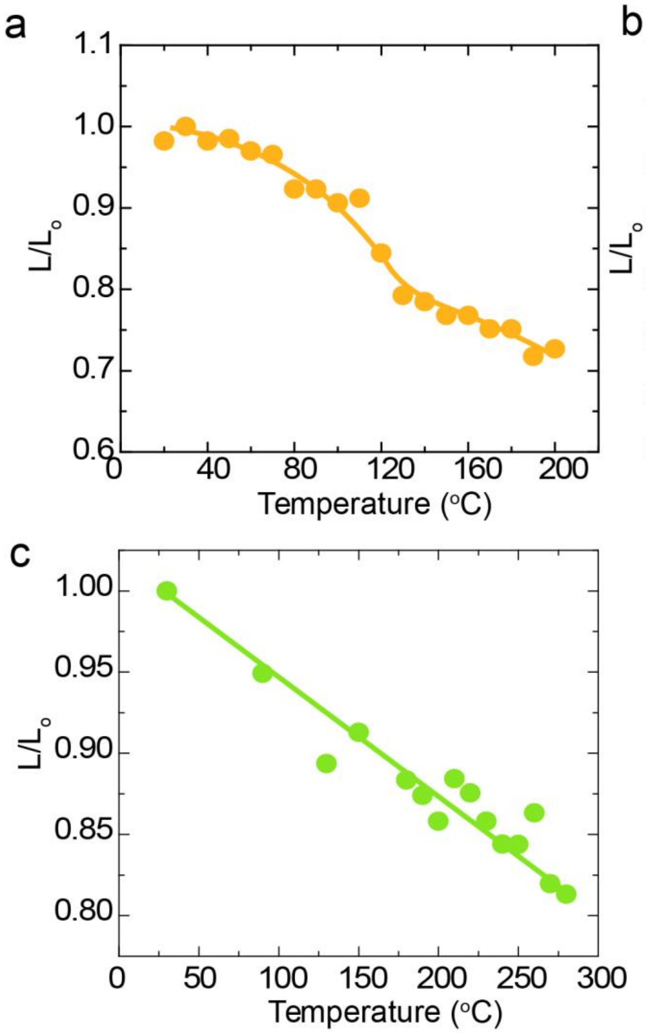

Supplementary Figure 5 | Mechanical testing of LCN actuators. Thermally induced contraction in **(a)** side-chain LCN for contracting-expanding deformation mode, **(b)** main-chain LCN for freestyle deformation and **(c)** side-chain LCN for bending and twisting deformation modes. L: length of the deformed sample and L_o the original length.

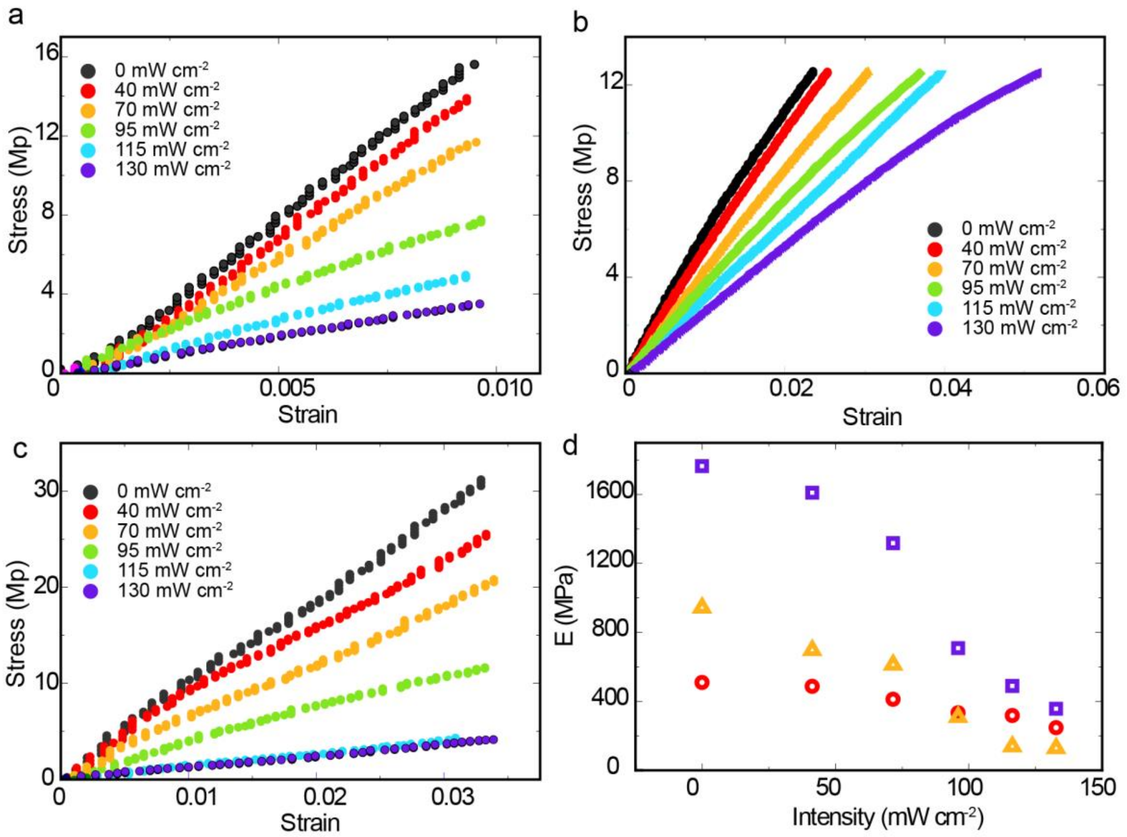

Supplementary Figure 6 | Stress−strain diagrams of LCNs upon different illumination. **(a)** Side-chain LCN with azobenzene crosslinkers for bending and twisting actuation. **(b)** Main-chain LCN for freestyle actuation. **(c)** Side-chain LCN with Disperse Red 1 for contracting-expanding actuation. The failure strain of side-chain LCNs in **(a)** and **(c)** is around 4 %. The failure stain of main-chain LCN **(b)** is typically about 20% at room temperature and > 60 % at an elevated temperature, e.g. 60 °C. **(d)** Young's modulus of LCNs upon different intensity. Squares: azobenzene crosslinked LCN, triangles: Disperse Red 1 doped LCN and circles: main-chain LCN. Irradiation: collimated LDE light source, centre wavelength 470 nm, full width at half maximum 20 nm. Note that, due to different photochemical/thermal mechanism and conversion efficiencies, a similar light intensity would trigger different elevated temperatures in different material systems. For thermally induced actuation, see Supplementary Figure 5.

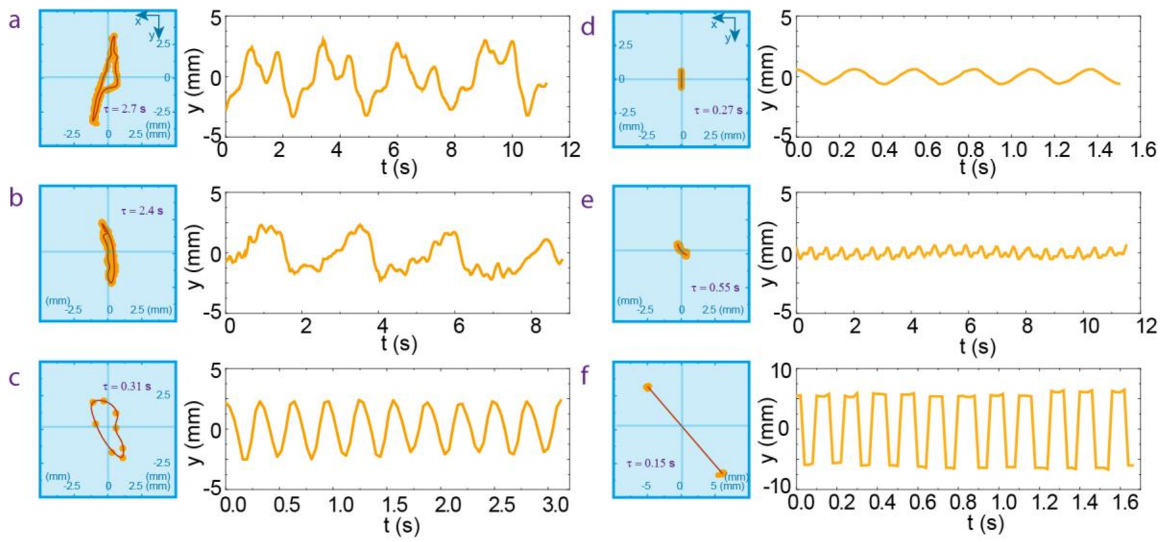

Supplementary Figure 7 | Different stabilized oscillation modes. **(a-f)** Observation of different short-term stabilized self-oscillation. Left panels: trajectory of tracking position on x-y plane. Right panels: time dependent displacement on y axis.

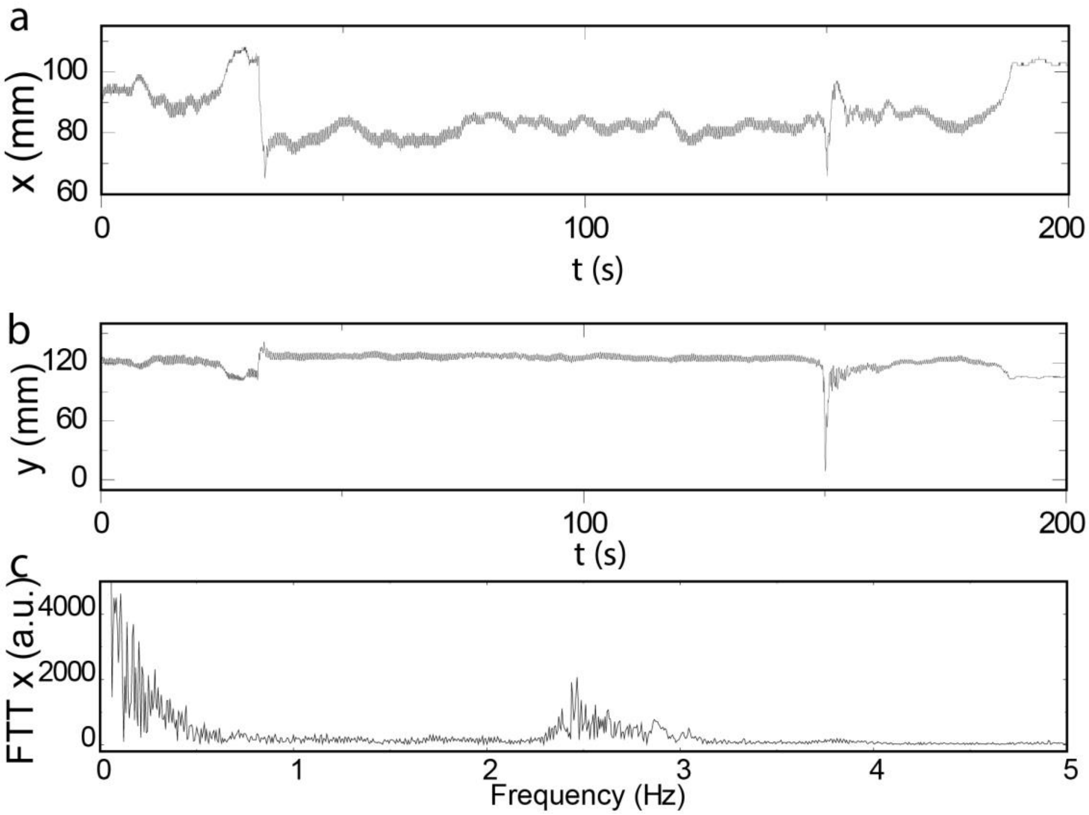

Supplementary Figure 8 | Evolution between modes. (a) Time dependent displacement of the tracking point of actuator on x axis. **(b)** Time dependent displacement of the same tracking spot on y axis. Fourier transform of **(a)**.

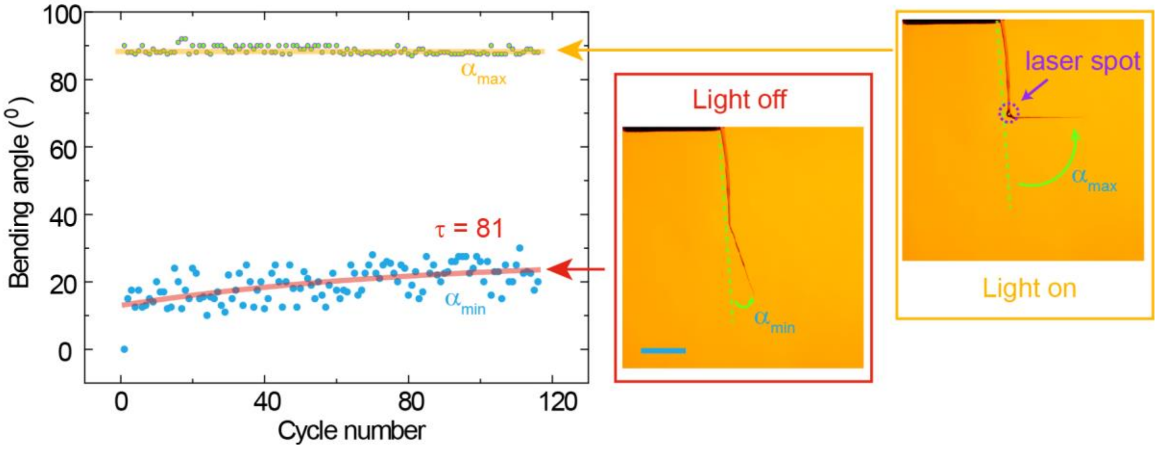

Supplementary Figure 9 | Irreversibility measurement in photomechanical actuation. The LCN actuator is exposed to cyclic laser excitation (488 nm, 100 mW), to reach maximum bending angle α_{max} upon light, and minimum bending angel α_{min} at the relax state (light off). Right: photograph of actuator upon light on/off condition. Scale bar is 5 mm.

Supplementary Methods.

Preparation.

Preparation of LCN actuator with bending and twisting deformation modes.

The LCN actuators are made by photopolymerization of a mixture containing 53 mol % of LC monomer 4-Methoxybenzoic acid 4-(6-acryloyloxyhexyloxy)phenyl ester (Synthon Chemicals), 18 mol % of LC monomer 4[4[6-Acryloxyhex-1-yl)oxyphenyl]carboxybenzonitrile (Synthon Chemicals), 22 mol % of di-acrylate crosslinker 1,4-Bis-[4-(6-acryloyloxyhexyloxy)benzoyloxy]-2-methylbenzene (Synthon Chemicals), 6 mol % of azo crosslinker 4,4'-Bis[9-(acryloyloxy)nonyloxy]azobenzene (Synthon Chemicals), and 1 mol % of photoinitiator Bis(2,4,6-trimethylbenzoyl)-phenylphosphineoxide (Sigma Aldrich). Chemical structures of all the compounds are shown in Supplementary Figure 10.

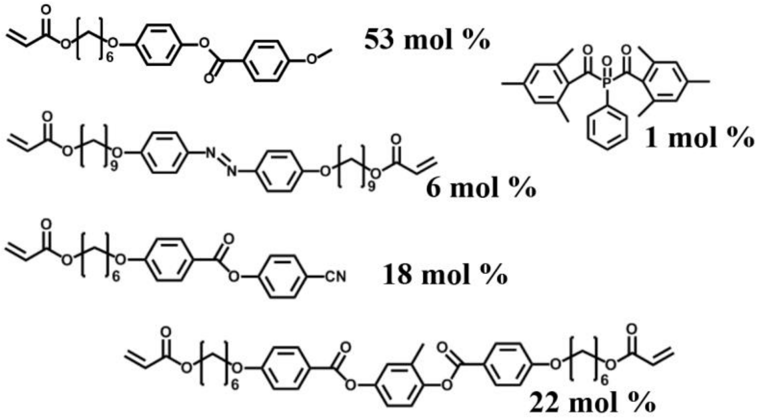

Supplementary Figure 10 | Chemical structures. Chemical structures of molecules used in actuators with bending and twisting modes.

All molecules were used as received. The monomer mixture was dissolved in dichloromethane (DCM) and filtered through PTFE syringe filter (Sigma Aldrich, pore size 0.2 µm), after which it was stirred at 80 °C (100 RPM) for 3 h in order to remove the solvent. For cell preparation, glass substrates were cleaned by successive sonication in 2-propanol and acetone

baths (20 min each), and dried under the flow of nitrogen gas. Two glass slides were spin coated with 1 wt% water solution of polyvinyl alcohol (PVA, Sigma Aldrich; 4000 RPM, 1 min) and rubbed uni-directionally by using a satin cloth. After rubbing, the PVA substrates were blown with high-pressure nitrogen to remove dust particles from the surfaces. Two PVA coated substrates were fixed together with UV glue (UVS 91, Norland Products INC., Cranbury, NJ) using spacer particles (Thermo scientific, 50 μm) to define the cell thickness. Rubbing directions on both substrates are paralleled in order to achieve a planar alignment. The monomer mixture was then infiltrated into the cell on a heating stage at 90 °C and cooled down to 50 °C with a rate of 5 °C min^{-1}. An LED (Prior Scientific; 420 nm, 11 mW cm^{-2}, 30 min) was used to polymerize the LC mixture. The cell was opened, and strip-like LCNs were cut out from the film by using a blade. For bending actuation mode, the strip (5.5×1.5×0.05 mm^3) was cut along the rubbing direction, while for the twisting mode, a 3.5×3.5×0.05 mm^3 sized film was cut.

Preparation of LCN actuator with contracting-expanding mode.

The LCN actuators were made by photopolymerization of a mixture containing 88.5 mol % of LC monomer 2-(((6-(acryloyloxy)hexyl)oxy)carbonyl)-1,4-phenylene bis(4-butoxybenzoate), 10 mol % of di-acrylate crosslinker 1,4-Bis-[4-(6-acryloyloxyhexyloxy)benzoyloxy]-2-methylbenzene (Synthon Chemicals), 0.5 mol % Disperse Red 1, N-Ethyl-N-(2-hydroxyethyl)-4-(4-nitrophenylazo)aniline (Sigma Aldrich), and 1 mol % of photoinitiator Bis(2,4,6-trimethylbenzoyl)-phenylphosphineoxide (Sigma Aldrich). The Chemical structures are shown in Supplementary Figure 11. The LC monomers are synthesized following the procedures of Ref. 1, other molecules were used as received.

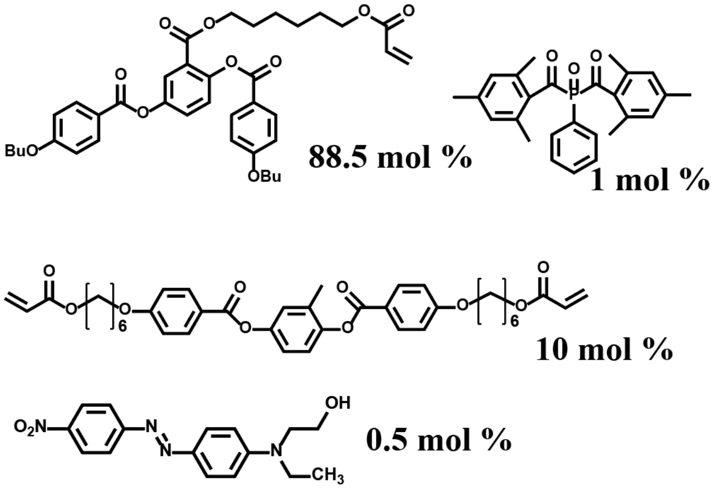

Supplementary Figure 11 | Chemical structures. Chemical structures of molecules used in actuator with contracting-expanding mode.

The monomer mixture was stirred at 80 °C (100 RPM) for 3 h, before infiltrating into a cell on a heating stage at 90 °C. The cell consisted of two parallel rubbed substrates coated with PVA, and spacer particles (Thermo scientific, 100 µm) in between to define the cell thickness. Soon after the infiltration, the cell was brought under a laser engraver (FLUX Delta+), to polymerize LCN linear structures using laser scanning along the rubbing direction (405 nm, 30 mW, focus spot: about 100 µm). The cell was opened by using a blade, the unpolymerized monomers were washed away by two baths of 2-propanol at 60 °C (5 min each). Then, the fibre like LCN structure (about 100 µm in diameter, 1 cm long) was removed by using a blade.

Preparation of LCN actuator with freestyle modes.

A two-step *in-situ* acyclic diene metathesis polymerization/crosslinking approach was used, following the procedure of Ref. 2. The LCN is composed of 89.84 mol% of α, ω -diene LC monomer 4-undec-10-enyloxy-benzoic acid 4-dec-9-enyloxy-phenyl ester, 8.82 mol% of four-alkenyl-armed crosslinker 2,5-bis[(1-dec-9-enyl-undec-10-neyl-4-carboxylate-piperidyl-amino) thiophenyl] croconium and 1.34 mol% of Grubbs second generation catalyst. The chemical

structures of molecules in used are presented in Supplementary Figure 12.

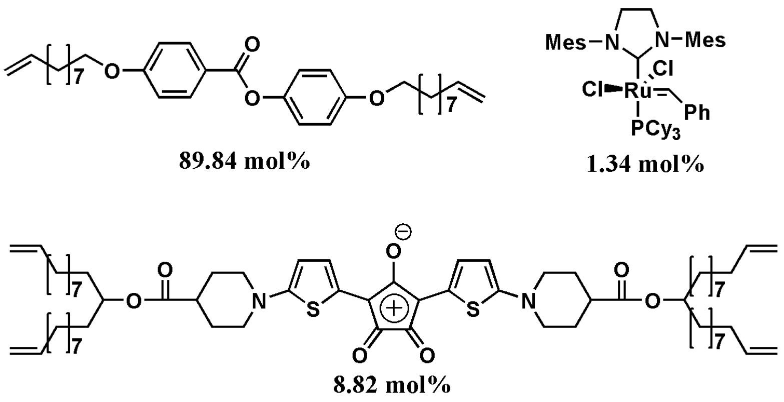

Supplementary Figure 12 | Chemical structures. Chemical structures of molecules used in actuator with freestyle deformation modes.

A mixture containing LC monomer, crosslinker and Grubbs second generation catalyst was dissolved in fresh anhydrous toluene and mixed under sonication for 2 min. The first step of polymerization/crosslinking process was conducted at 60 °C for 3 h in a 50 mL flat-bottomed flask equipped with a nitrogen flow, resulting in a partially crosslinked polydomain LC gel. Then the gel was carefully peeled off from the flask by using a tweezer, and cut into strips by a blade. An external loading was exerted to stretch the LC gel strip, in order to align the mesogens along the stretching direction. The stretched strip was fixed on a glass slide by two binder clips and kept in a nitrogen-filled oven for 2 days at 120 °C to reach a fully crosslinking of the material. The fully crosslinked sample was cut into small strips, with typical dimension of 3 mm width, 25 – 40 mm long, and 100 – 120 μm in thickness.

Photomechanical actuation and oscillation in basic deformation modes.

Bending and twisting modes.

An unfocused continuous 488 nm laser beam (180 mW) was incident onto the surface of

an LCN cantilever, of which one end was mechanically fixed on a stage, while the other end could be freely moving under illumination. Upon 488 nm laser excitation, azo crosslinkers inside LCN can repeatedly isomerize between trans and cis states, together with a significant contribution of photothermal effect within laser area the LCN cantilever is able to deform toward the light source. Such photoactuation mechanism was used to induce self-oscillation with bending and twisting modes, as drawn in **Supplementary Figure 13a**. For a bending mode, laser beam first hit one side of the LCN, leading to a bending toward the opposite side. As far as the cantilever bent, it blocked the illumination from the previous surface, instead, the laser beam started hitting the opposite side, forcing the cantilever deflect back to the original orientation. Once the first surface exposed to laser beam again, it bent back as before and a new cycle of movement started. Twisting mode has similar situation as the bending one. However, the strip was cut perpendicularly to the planar alignment, with one end mechanically fixed while the end could only twist upon laser excitation. Under illumination from the side direction, upper surface might face the light and absorb light energy that would subsequently cause the deformation. After twisting, the bottom surface was upon illumination while the upper one turned into the darkness. Soon after twisting backward, the upper surface experienced the same illumination again, and oscillating cycles started (**Supplementary Figure 13, b**).

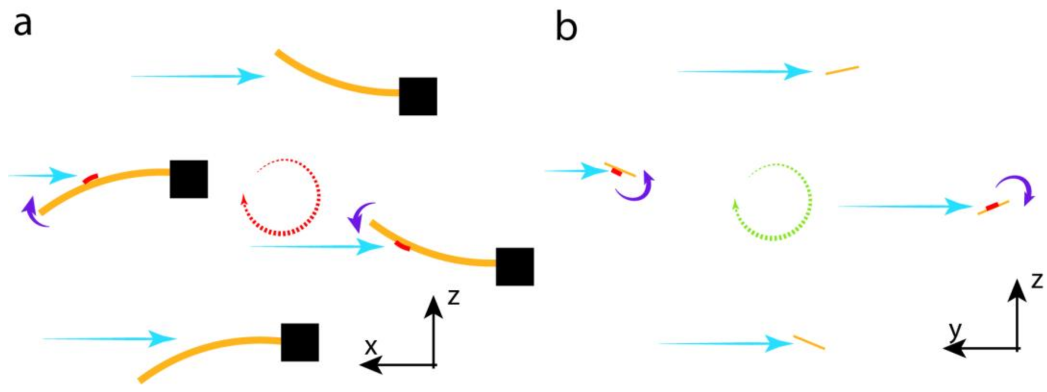

Supplementary Figure 13 | Principle of light-fueled oscillation. Light induced self-oscillating cycles in bending **(a)** and twisting deformation modes **(b)**.

Contracting-expanding mode.

The LCN used for this mode is a purely thermally responsive actuator. A piece of sample about 1 mm long was fixed on a stage at one end, while the other end was excited under a 488 nm laser beam (100 mW) focused by an objective (10 ×, NA=0.25). Light was absorbed by LCN at a confined area, then the energy was transferred to heat and conducted along the cantilever to the rest of the actuator body. A larger portion of the cantilever was actuated, leading to a contraction that moved the edge out of the laser spot. Material cooled down in the dark, and relaxed back to reach the same spot again, then being heated up. Therefore, a cyclic contracting-expanding oscillator was realized. The schematic drawing of oscillation process is shown in **Supplementary Figure 14**.

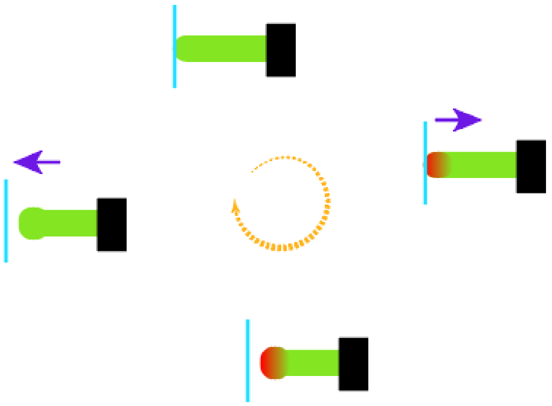

Supplementary Figure 14 | Principle of light-driven oscillation. Light induced self-oscillating cycles in contracting-expanding mode.

Natural frequency calculations.

Natural frequency for a bending cantilever.

For the transverse vibrations of a cantilever beam without damping, the natural frequencies

can be described by equation as[3],

$$\omega_n = \beta_n^2 \sqrt{\frac{EI}{\rho A}}. \quad (1)$$

ρ =1.2×10 g cm^{-3}, is the material density. β_n are eigenvalues by solving cantilever beam eigenfunctions, describing the nth harmonic oscillation. For a basic bending mode, $\beta_0 = 1.8751/L$, where $L = 5\ mm$ is the free-moving length of the cantilever. E is the Young's modulus, estimated to be 100 MPa upon laser excitation during oscillating. I is second moments of area, $I = \frac{w\times d^3}{12}$, where $w = 1.5\ mm$ is the width, and $d = 0.05mm$ is the thickness. A is the cross-section, equals to w×d. Herein, we got a basic bending oscillation mode with frequency $f = \omega/2\pi$ at 92 Hz.

Natural frequency for contracting-expanding cantilever.

For the longitudinal vibrations in a fixed-free rod, the basic mode vibrations can be described by equation as[3],

$$\omega = \frac{1}{2}\frac{\pi c}{L}, \quad (2)$$

where $c = \sqrt{\frac{E}{\rho}}$, Young's modulus, E is estimated to be around 10 MPa upon irradiation, density ρ = 1.2 g cm^{-3}. L, length of actuator, 1 mm. The oscillation frequency is calculated to be 23 kHz.

Natural frequency for twisting mode.

The basic torsional vibration in a fixed-free cantilever with L length, can be considered as a equivalence of half portion oscillation in a $2L$ long free-free cantilever, where the frequency can be described by equations as[4],

$$f = \frac{c_T}{8L}, \quad (3)$$

$$c_T = \sqrt{\frac{GK}{\rho I_p}}, \tag{4}$$

$$G = \frac{E}{2(1+v),} \tag{5}$$

$$K = \frac{wd^3}{16}[\frac{16}{3} - 3.36\frac{d}{w}(1 - \frac{d^4}{12w^4})], \tag{6}$$

$$I_p = \frac{wd}{12}(w^2 + d^2). \tag{7}$$

Sample dimension: width w, 3.5 mm, leng L, 3.5 mm and thickness d, 0.05 mm. Young's modulus E is estimated to be 100 MPa under laser excitation. Poisson's ratio $v = 0.5$. Density of material $\rho = 1.2$ g cm^{-3}. The twisting frequency is calculated to be 169 Hz.

Data collection.

Oscillation videos were recorded using a Canon 5D Mark III camera with 100 mm lens (50 fps) or Sony RX100V high-speed camera (1000 fps). For tracking the oscillating position, fluorescence particles (Rhodamine 6G, Merck) were put at the end of LCN actuator, as an indicator during oscillation. For this, few milligrams of fluorescence dyes were mixed with UV glue (UVS 91, Norland Products Inc., Cranbury, NJ), and dropped on to the end of LCN strip follow up with a UV polymerization. A weak UV source (0.1 mW cm^{-2}) was used to illuminate the whole sample, and the fluorescence images were captured by using the camera equipped with an optical filter cutting off wavelength < 500 nm. The tracking position was analysed with a video analysis software (Kinovea).

Supplementary References

1. Keller, P., Thomsen, D. L. & Li, M.-H. Facile and inexpensive synthesis of α,β,β'-deuterated liquid crystalline and classical acrylate monomers. *Macromolecules* **35**, 581-584 (2002).

2. Liu, L., Liu, M. H., Deng, L. L., Lin, B. P. & Yang, H. Near-infrared chromophore functionalized soft actuator with ultrafast photoresponsive speed and superior mechanical property. *J. Am. Chem. Soc.***139**, 11333–11336 (2017).

3. Rao, S. S. Vibration of Continuous Systems (Wiley, New York, 2007)

4. https://www.acs.psu.edu/drussell/Demos/Torsional/torsional.html
