# Supplementary material for: Light-fuelled freestyle self-oscillators
Source: Nat Commun. 2019 Nov 7;10:5057. doi: 10.1038/s41467-019-13077-6 (PMC6838320; doi:10.1038/s41467-019-13077-6)
Supplement: Supplementary file 3 — Description of Additional Supplementary Files [file 41467_2019_13077_MOESM3_ESM.pdf]

Description of Additional Supplementary Files

File Name: Supplementary Movie 1
Description: **Slow motion video for light-fuelled self-oscillator based on bending deformation mode.** A planar-aligned liquid crystal network actuator with 5.5×1.5×0.05 mm3 size is mechanically fixed on a stage. The self-oscillation is induced by excitation of 488 nm laser beam (180 mW) propagating along the alignment/strip direction. The movie takes place in 40× slower speed.

File Name: Supplementary Movie 2
Description: **Light-fuelled self-oscillator with contraction-expansion mode. A liquid crystal elastomer fibre with planar alignment is fixed on a stage.** A laser beam is focused on the tip of the actuator (100 mW, focused by 10× objective, NA= 0.25) to induce self-oscillation. By changing the laser spot position (while maintaining the same power), different kinds of oscillations are obtained. During each stable oscillation, the laser beam is not spatially or temporally modified. The movie takes place in real time.

File Name: Supplementary Movie 3
Description: **Light-fuelled self-oscillator with twisting mode.** A planar-aligned liquid crystal network actuator (3.5×3.5×0.05 mm3) is mechanically fixed on a stage. The laser is propagating 2 (2) E-Mail: arri.priimagi@tuni.fi | Tel. +358 (0) 44 515 0300 | research.tuni.fi/spm on the side direction (488 nm, 180 mW), along with the molecular alignment. The movie takes place in real time.

File Name: Supplementary Movie 4
Description: **Different oscillation modes obtained in a freestyle oscillator.** A main-chain liquid crystal elastomer actuator (size: 3×25×0.1 mm3) is hung on a human hair through a punctured hole. A 488 nm laser beam (160 mW) is incident onto different positions of the actuator to induce different oscillation modes. During each stable oscillation, the laser beam is not spatially or temporally modified. The movie takes place in real time.

File Name: Supplementary Movie 5
Description: **A self-oscillator dancing on a human hair.** A main-chain liquid crystal elastomer actuator (size: 2×20×0.1 mm3) is hung on a human hair through a hole. By changeing the laser spot position (488 nm 160 mW), the oscillator rapidly switches between different oscillation modes, performing a dancing-like movement on the human hair. The movie takes place in real time.
